# Emerging Cellular Therapies for Glioblastoma Multiforme

**DOI:** 10.7759/cureus.2305

**Published:** 2018-03-11

**Authors:** Paul J Choi, R. Shane Tubbs, Rod J Oskouian

**Affiliations:** 1 Clinical Anatomy, Seattle Science Foundation; 2 Neurosurgery, Seattle Science Foundation; 3 Neurosurgery, Swedish Neuroscience Institute

**Keywords:** glioblastoma multiforme, cellular therapy, temozolomide, molecular envelope technology, cellular vehicle, oncolytic virus

## Abstract

Glioblastoma multiforme (GBM) is the most common type of malignant primary brain cancer in adults. It is composed of highly malignant cells that display metastatic and angiogenic characteristics, making it resistant to current first-line chemotherapy with temozolomide, an alkylating agent. Despite many years of research, GBM remains poorly responsive to multiple available therapies, giving GBM patients, who receive the conventional combination of chemoradiotherapies and surgical resection, a dismal prognosis. There is growing evidence that the conventional systemic chemotherapeutic agents for GBM are ineffective in improving the disease progression. We aim to explore the emerging cellular therapies which may play a significant role in treating GBM.

## Introduction and background

Glioblastoma multiforme (GBM) is the most common type of malignant primary brain cancer in adults [1-3]. It is composed of highly malignant cells that display metastatic and angiogenic characteristics, making it resistant to current first-line chemotherapy with temozolomide, an alkylating agent [1,3]. A subtype of GBM, which is resistant to both temozolomide and radiotherapy, has also been described in the literature [1]. The mechanism of resistance in this subtype has been attributed to mutation of the deoxyribonucleic acid (DNA) repair enzymes [1]. Despite many years of research, GBM remains poorly responsive to available therapies [2,4-5]. Therefore, GBM patients who receive the conventional combination of chemoradiotherapies and surgical resection still have a dismal average one-year survival and a high recurrence rate [2-5]. One of the main difficulties in treating GBM is getting a significant dosage of the chemotherapeutic agent to cross the blood-brain barrier (BBB) and reach the lesion while limiting its severe adverse systemic effects [4].

## Review

Flaws in conventional chemotherapy

A series of recent in-vitro and in-vivo trials have demonstrated the efficacy of nitrosourea compounds, such as fotemustine, in recurrent human GBM cell lines [5]. This prodrug, which becomes activated into a lipophilic product by non-enzymatic hydroxylation in the liver, can freely cross the BBB [5]. However, it carries a 30% risk of causing severe hematological toxicity, so it is suggested only as the second-line treatment for recurrent GBM [5]. It is likely that the first-line agent for GBM, temozolomide, also carries a risk of dose-limiting bone marrow suppression [3]. Moreover, Houshyari et al. concluded that systemic chemotherapy has no significant survival benefit for patients with GBM [6].

Emerging technologies to deliver tumoricidal agents across the blood-brain barrier

In order to bypass the detrimental hematotoxic effects of the current systemic chemotherapeutic regimen, many promising technologies have recently emerged, including the direct placing of Gliadel wafers (Arbor Pharmaceuticals, Atlanta, Georgia, US), a set of biological polymer discs containing carmustine, a nitrosourea, within the brain following tumor resection, and Molecular Envelope Technology (MET) nanoparticles [3,7-9].

In recent years, Fisusi et al. have demonstrated that MET nanoparticles have significant efficacy in exposing GBM cells to a higher dosage of chemotherapeutic agents while limiting the myelosuppressive side effects in a mouse model [3]. These authors enveloped the chemotherapeutic agents in an engineered nanoparticle containing various amounts of hydrophilic and hydrophobic components [10]. In principle, this technology allows the drug to be deposited only within the brain, bypassing the bone marrow.

A similar concept relates to the use of stem cells that can cross the BBB as vehicles for delivering drugs to the brain. The efficacy of mesenchymal and neural stem cells as drug delivery vectors has recently been explored by in-vitro studies and in-vivo animal studies and has yielded promising results [4]. For instance, Mariotti et al. proposed the efficacy of drug-containing mesenchymal stem cells administered intranasally through the cribriform plate, causing satisfactory tumor regression in a mouse model [4]. Moreover, researchers have used a similar delivery method to expose the GBM lesion to oncolytic viruses and genes that induce selective apoptosis in the malignant cells [4]. For example, Cheema et al. obtained a promising result from studying the effect of genetically engineered herpes simplex virus in reducing angiogenesis in a mouse model [11]. Other oncolytic viruses, such as adenoviruses and retroviruses, have proved to be efficacious and are currently being studied in clinical trials [4]. For example, an ongoing phase 1 study entitled "Neural Stem Cell-Based Virotherapy of Newly Diagnosed Malignant Glioma” by the Northwestern University (ClinicalTrials.gov ID: NCT03072134) is assessing the effectiveness of loading neural stem cells with oncolytic adenovirus and using them as a delivery vehicle to the brain. This work is based on the authors’ preclinical studies, which showed satisfactory tumor regression without additional toxicities.

## Conclusions

Although researchers have struggled for many years to improve the survival outcome of GBM patients, the prognosis of the disease seems to have remained unchanged over the last six decades despite advances in modern combination therapies. Although systemic chemotherapeutics are demonstrably efficacious in destroying the malignant cells, the amount that reaches the BBB is limited owing to the danger of significant systemic toxicity. This led scientists to explore novel methods of delivery including direct insertion of drug-containing discs into the brain parenchyma, use of engineered nanoparticles as a drug delivery vehicle, and deployment of stem cells to carry drugs and genetically engineered oncolytic viruses to the brain in order to bypass the severe hematological side effects. Promising in-vitro studies that have explored the biochemistry of alkylating agents, nitrosourea, and oncolytic viruses, and the use of stem cells as a vector to deliver the agents, have been translated into in-vivo mouse model studies (since mice have similar genetics to humans, a precise biochemistry and histopathology of genetic mutation in the human brain can be simulated in theirs; moreover, mice have a short life cycle that allows researchers to study many generations). The knowledge retrieved from mouse models is subsequently translated into clinical trials to test the technology’s effect on the human body.
